# Clusters of SARS-CoV-2 Lineage B.1.1.7 Infection after Vaccination with Adenovirus-Vectored and Inactivated Vaccines

**DOI:** 10.3390/v13112127

**Published:** 2021-10-22

**Authors:** William M. de Souza, Stéfanie P. Muraro, Gabriela F. Souza, Mariene R. Amorim, Renata Sesti-Costa, Luciana S. Mofatto, Julia Forato, Priscilla P. Barbosa, Daniel A. Toledo-Teixeira, Karina Bispo-dos-Santos, Pierina L. Parise, Natalia S. Brunetti, Joselia C. O. Moreira, Vitor A. Costa, Daniela M. Cardozo, Maria L. Moretti, Silvia Barros-Mazon, Gabriela F. Marchesi, Christiane Ambrosio, Fernando R. Spilki, Valeria C. Almeida, Andre S. Vieira, Lair Zambon, Alessandro S. Farias, Marcelo Addas-Carvalho, Bruno D. Benites, Rafael E. Marques, Ester C. Sabino, Andrea B. Von Zuben, Scott C. Weaver, Nuno R. Faria, Fabiana Granja, Rodrigo N. Angerami, José Luiz Proença-Módena

**Affiliations:** 1 Virology Research Centre, Ribeirão Preto Medical School, University of São Paulo, Ribeirão Preto 14049-900, Brazil; wmdesouz@utmb.edu; 2 Department of Microbiology and Immunology, University of Texas Medical Branch, Galveston, TX 77555, USA; sweaver@utmb.edu; 3 Laboratory of Emerging Viruses, Department of Genetics, Microbiology and Immunology, Institute of Biology, University of Campinas, Campinas 13083-862, Brazil; stefaniemuraro@gmail.com (S.P.M.); gabriela.sfabiano@gmail.com (G.F.S.); mariene.ramorim@gmail.com (M.R.A.); luciana.mofatto@gmail.com (L.S.M.); foratojulia@gmail.com (J.F.); priscillapaschoal@hotmail.com (P.P.B.); teixeiradatt@gmail.com (D.A.T.-T.); karina.bsantos@hotmail.com (K.B.-d.-S.); pierinalp@gmail.com (P.L.P.); fabi.granja@yahoo.com.br (F.G.); 4 Brazilian Biosciences National Laboratory, Brazilian Centre for Research in Energy and Materials, Campinas 13083-100, Brazil; renata.sesti@gmail.com (R.S.-C.); rempsufmg@gmail.com (R.E.M.); 5 Hematology and Hemotherapy Center, University of Campinas, Campinas 13083-862, Brazil; vitorc@unicamp.br (V.A.C.); maddas@unicamp.br (M.A.-C.); benites@unicamp.br (B.D.B.); 6 Autoimmune Research Laboratory, Department of Genetics, Microbiology and Immunology, Institute of Biology, University of Campinas, Campinas 13083-862, Brazil; nbrunetti.bio@gmail.com (N.S.B.); asfarias@unicamp.br (A.S.F.); 7 Department of Structural and Functional Biology, Institute of Biology, University of Campinas, Campinas 13083-862, Brazil; jocris@unicamp.br (J.C.O.M.); vieira.as@gmail.com (A.S.V.); 8 Department of Clinical Pathology, School of Medical Sciences, University of Campinas, Campinas 13083-862, Brazil; danielamcardozo@hc.unicamp.br (D.M.C.); sbmazon@unicamp.br (S.B.-M.); 9 Department of Internal Medicine, School of Medical Sciences, University of Campinas, Campinas 13083-862, Brazil; lmoretti@unicamp.br (M.L.M.); lzambon@fcm.unicamp.br (L.Z.); rodrigo.angerami@gmail.com (R.N.A.); 10 Campinas Department of Public Health Surveillance, Campinas 13015-904, Brazil; gabriela.marchesi@campinas.sp.gov.br (G.F.M.); christiane.ambrosio@campinas.sp.gov.br (C.A.); valcalmeida@gmail.com (V.C.A.); andreabrunovonzuben@gmail.com (A.B.V.Z.); 11 One Health Laboratory, Feevale University, Novo Hamburgo 93510-235, Brazil; fernandors@feevale.br; 12 Obesity and Comorbidities Research Center, University of Campinas, Campinas 13083-862, Brazil; 13 Experimental Medicine Research Cluster, University of Campinas, Campinas 13083-862, Brazil; 14 Tropical Medicine Institute, Medical School, University of São Paulo, São Paulo 05403-907, Brazil; sabinoec@gmail.com; 15 Department of Infectious and Parasitic Disease, Medical School, University of São Paulo, São Paulo 05403-000, Brazil; n.faria@imperial.ac.uk; 16 Institute for Human Infection and Immunity, University of Texas Medical Branch, Galveston, TX 77555, USA; 17 Department of Zoology, University of Oxford, Oxford OX1 2JD, UK; 18 MRC Centre for Global Infectious Disease Analysis, J-IDEA, Imperial College London, London SW7 2AZ, UK; 19 Biodiversity Research Centre, Federal University of Roraima, Boa Vista 72000-000, Brazil

**Keywords:** SARS-CoV-2, COVID-19, variant of concern, B.1.1.7, vaccine, outbreak

## Abstract

A SARS-CoV-2 B.1.1.7 variant of concern (VOC) has been associated with increased transmissibility, hospitalization, and mortality. This study aimed to explore the factors associated with B.1.1.7 VOC infection in the context of vaccination. On March 2021, we detected SARS-CoV-2 RNA in nasopharyngeal samples from 14 of 22 individuals vaccinated with a single-dose of ChAdOx1 (outbreak A, *n* = 26), and 22 of 42 of individuals with two doses of the CoronaVac vaccine (outbreak B, *n* = 52) for breakthrough infection rates for ChAdOx1 of 63.6% and 52.4% for CoronaVac. The outbreaks were caused by two independent clusters of the B.1.1.7 VOC. The serum of PCR-positive symptomatic SARS-CoV-2-infected individuals had ~1.8–3.4-fold more neutralizing capacity against B.1.1.7 compared to the serum of asymptomatic individuals. These data based on exploratory analysis suggest that the B.1.1.7 variant can infect individuals partially immunized with a single dose of an adenovirus-vectored vaccine or fully immunized with two doses of an inactivated vaccine, although the vaccines were able to reduce the risk of severe disease and death caused by this VOC, even in the elderly.

## 1. Introduction

*Severe acute respiratory syndrome coronavirus 2* (SARS-CoV-2) lineage B.1.1.7 (also known as the alpha variant) was first detected in the UK in late 2020. To date, this lineage has been reported in 163 countries [1]. In March 2021, B.1.1.7 became the dominant lineage in the UK, USA, Denmark, and Switzerland [2]. B.1.1.7 features 17 mutations and three deletions, including the N501Y substitution in the spike protein [2]. In some studies, this lineage has been associated with enhanced transmissibility, mortality, and more coronavirus disease 19 (COVID-19) hospitalizations compared to previously circulating SARS-CoV-2 lineages [2,3,4]. In addition, in vitro studies have shown a reduction of up to 11.4-fold in the neutralizing antibody capacity of plasma of vaccinated individuals (i.e., BNT162b2, mRNA-1273, and ChAdOx1) against wild-type B.1.1.7 isolates or pseudoviruses featuring the B.1.1.7 spike mutations, which suggests immune escape [5,6,7]. However, other studies show little or no difference in the neutralization antibody capacity of the plasma of vaccinated individuals against the B.1.1.7 variant compared to the original Wuhan or equivalent strains [8,9].

In Brazil, the first cases caused by B.1.1.7 lineage were identified in São Paulo city in patients who traveled from the UK in December 2020 [10]. The B.1.1.7 lineage has recently been detected in over 10 Brazilian states resulting from multiples introductions [11]. In January 2021, the CoronaVac (Sinovac) and ChAdOx1 (Oxford-AstraZeneca) vaccines received Emergency Use Authorization from the Ministry of Health of Brazil. Both vaccines require two doses for completion of the vaccination series. The recommended interval between doses is 14–28 days for CoronaVac and 90 days for the ChAdOx1 vaccine [12,13]. As of 27 August 2021, 47.5% (57.4 of 120.8 million) of individuals in Brazil had received a single dose of the ChAdOx1 vaccine, while 28.6% (34.6 of 120.8 million) were CoronaVac recipients. On the other hand, 47.1% (25.6 of 54.4 million) of individuals were immunized with the complete series (i.e., two doses) of CoronaVac, and 41.2% (22.4 of 54.4 million) received two doses of the ChAdOx1 vaccine.

To increase the number of individuals receiving at least a single dose of vaccine to avert COVID-19 severity and prevent mortality, it has been proposed to delay the second dose of the primary immunization series [14,15,16,17,18]. Several studies have investigated whether a single-dose regimen of the adenovirus-vectored vaccine or full immunization with an inactivated vaccine is sufficient to interrupt SARS-CoV-2 transmission [19,20,21]. This study, therefore, aimed to evaluate factors associated with two B.1.1.7 transmission clusters in the context of vaccination with ChAdOx1 and CoronaVac vaccines.

## 2. Materials and Methods

### 2.1. Study Design, Participants, and Ethics

The SARS-CoV-2 outbreak investigations were performed in the collaboration with the Department of Health Surveillance of Campinas city in a convent and a long-term care (LTC) facility in Campinas city, São Paulo State, Brazil. Inclusion criteria for our cohorts were individuals at least 18 years of age exposed to residents infected with SARS-CoV-2 in these two locations in March 2021. Residents and employees from both locations were included in the study. Nasopharyngeal and serum specimens were collected during a visit of health surveillance assistants between 24 and 29 March 2021, after a positive laboratory test in a resident and the onset of symptoms in other residents. Clinical data were collected from electronic medical records, including age, sex, symptom duration, the time between symptoms and collection, vaccination date, and hospitalization during the SARS-CoV-2 infection (Table S1). The collection of biological samples took place in the context of epidemiological investigation of the COVID-19 outbreak, including laboratory investigation of symptomatic cases and exposed contacts conducted by the municipal health surveillance department. 

### 2.2. RNA Extraction and Real-Time Quantitative Polymerase Chain Reaction

Viral RNA was extracted from the nasopharyngeal swab samples using the Quick-DNA/RNA viral kit (Catalog number: D7021, Zymo Research, Irvine, CA, USA) according to the manufacturer’s instructions. The RNA from samples was tested by real-time quantitative polymerase chain reaction (RT-qPCR) targeting the envelope gene [22], as well as the GeneFinder COVID-19 Plus RealAmp Kit (Catalog number: IFMR-45, OSANG Healthcare, Seoul, Korea). A confirmed SARS-CoV-2 case was defined as positive by RT-qPCR for at least one protocol. For comparing viral loads between ChAdOx1- and CoronaVac-vaccinated patients, we used nonparametric Mann–Whitney U test analysis. A Dunn–Bonferroni post hoc correction was applied. We conducted this statistical analysis using RStudio (v1.3.1) and considered statistical significance as a *p*-value < 0.05.

### 2.3. SARS-CoV-2 Genome Sequencing

Positive samples by RT-qPCR with cycle threshold (Ct) values <30 were submitted for SARS-CoV-2 genome sequencing carried out using the ARTIC network SARS-CoV-2 v3 primer scheme (https://artic.network/ncov-2019, accessed on 24 March 2020) protocol with the MinION platform (Oxford Nanopore Technologies, Oxford, UK) [23,24]. Then, FAST5 files containing the raw signal data were base-called, demultiplexed, and trimmed using Guppy version 4.4.1 (Oxford Nanopore Technologies, Oxford, UK). The reads were aligned against the reference genome Wuhan-Hu-1 (GenBank accession no. MN908947.3) using minimap2 version 2.17.r941 [25] and converted to a sorted BAM file using SAMtools [26]. Length filtering, quality testing, primmer trimming, variant calling, and consensus sequences were performed for each barcode using guppyplex (https://artic.network/ncov-2019, accessed on 24 March 2020). Genome regions with a depth <20-fold were represented with N characters. 

### 2.4. Genomic and Phylogenetic Analysis

Sequences with ≥75% genome coverage were uploaded to the CoV-GLUE online resource (http://cov-glue.cvr.gla.ac.uk/ accessed on 10 May 2021) and Pangolin COVID-19 Lineage Assigner tool (v.2.2.1, 25 May 2021) for mutation identification and lineage classification [27,28]. To contextualize newly sequenced genomes, we compiled a dataset of 255 B.1.1.7 sequences from Brazil and abroad (e.g., UK, USA, Spain, Portugal, France, and Italy) available in the GISAID until 26 June 2021 (https://www.gisaid.org, accessed on 26 June 2021). Duplicate sequences were removed from the alignments. Tables describing publicly available data used, including GISAID ID, authors, and the submitting laboratory, can be found in Table S2. Multiple sequence alignment was carried out using MAFFT version 7.309 [29], and manual adjustment was conducted using Geneious Prime 2020.2.3. A maximum-likelihood (ML) tree was performed using a 29,521-nucleotide alignment after removing 3’ and 5’ untranslated regions. An ML tree was inferred using the IQ-TREE version 1.6.12, and a nucleotide substitution model was determined with ModelFinder [30,31]. In addition, our dataset was screened for recombination using RDP v.4 [32]. 

### 2.5. SARS-CoV-2 Virus Isolation

Virus isolation in Vero cells (CCL-81, ATCC, Manassas, VA, USA) was performed using nasopharyngeal lavage samples as previously described [33]. Subsequently, virus replication was confirmed in the culture supernatant by observing lower Ct values in RT-qPCR [22]. The first passage was propagated in Vero cells to obtain a greater volume stock. Both first and second passages were titrated by plaque assay. All experiments related to cell culture and viral replication were performed under biosafety level 3 (BSL-3) in the Laboratory of Emerging Viruses of the University of Campinas, Brazil. In addition, we also sequenced SARS-CoV-2 genomes from the first and second passages, as described above.

### 2.6. Serology 

Immunoglobulin G (IgG) antibodies against the spike receptor-binding domain (S-RBD) of SARS-CoV-2 in serum samples were measured by SARS-CoV-2 IgG II Quant (Catalog number: 06S6122, Alinity, Abbott Diagnostics, Chicago, IL, USA), according to the manufacturer’s instructions. This method is an automated chemiluminescent microparticle immunoassay (CMIA) used to detect IgG antibodies in a qualitative and quantitative form [34]. Results ≥ 50.0 AU/mL were considered positive [35]. Then, we compared IgG (S-RBD) antibody levels between individuals PCR-positive and -negative who were vaccinated with a single dose of ChAdOx1 or two doses of CoronaVac and those who were unvaccinated. For comparing between groups, we used nonparametric Kruskal–Wallis one-way analysis. A Dunn’s multiple comparison test correction was applied. We conducted this statistical analysis using RStudio (v1.3.1) and considered statistical significance as a *p*-value < 0.05. In addition, we measured the total SARS-CoV-2 antibodies (i.e., IgA, IgM, and IgG) specific against the SARS-CoV-2 nucleocapsid protein from ChAdOx1 recipients using the Elecsys Anti-SARS-CoV-2 electrochemiluminescence immunoassay (Catalogue number: 49025801, Roche Diagnostics, Rotkreuz, Switzerland) following the manufacturer’s instructions. Results are reported as numeric values in the form of a qualitative result as nonreactive (cutoff index (COI) < 1.0; negative) and reactive (COI ≥ 1.0; positive). 

### 2.7. Plaque Reduction Neutralization Test 

An in-house plaque reduction neutralization test (PRNT) was performed as previously described [33]. Plaque reduction was calculated for each sample by comparing the number of plaques in wells inoculated with SARS-CoV-2 lineage B.1.1.7 isolate. Mean serum neutralizing antibody titers (50% neutralization testing (PRNT_50_)) were calculated as an average of two independent experiments.

## 3. Results

**Figure 1:**
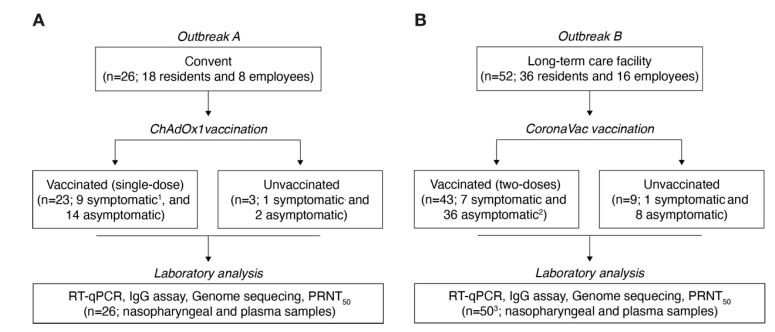
Flow diagram describing the outbreaks reported in this study. Outbreak A in convent (**A**), and outbreak B in long-term care facility (**B**). ^1^ Includes a nun who was vaccinated with Ad26.COV2.S (Janssen Pharmaceutical Companies, Beerse, Belgium). ^2^ Includes an employee vaccinated with a single dose of ChAdOx1. ^3^ Biological samples from a resident (ID: LTC20) and an employee (ID: LTC37) were not collected, the first because the patient was admitted to the hospital with COVID-19 and died 2 weeks later, and the second was paid time off during the collection samples time. Abbreviations: RT-qPCR; real-time quantitative polymerase chain reaction; IgG, immunoglobulin G; PRNT, plaque reduction neutralization tests.

In March 2021, we identified two COVID-19 outbreaks in vaccinated individuals who had received a single dose of ChAdOx1 in a convent (Outbreak A) or two doses of CoronaVac vaccine in an LTC facility (Outbreak B) in Campinas city, São Paulo State, Brazil (Figure 1). The vaccination itself was not associated with any serious adverse events in both cohorts.

**Figure 2:**
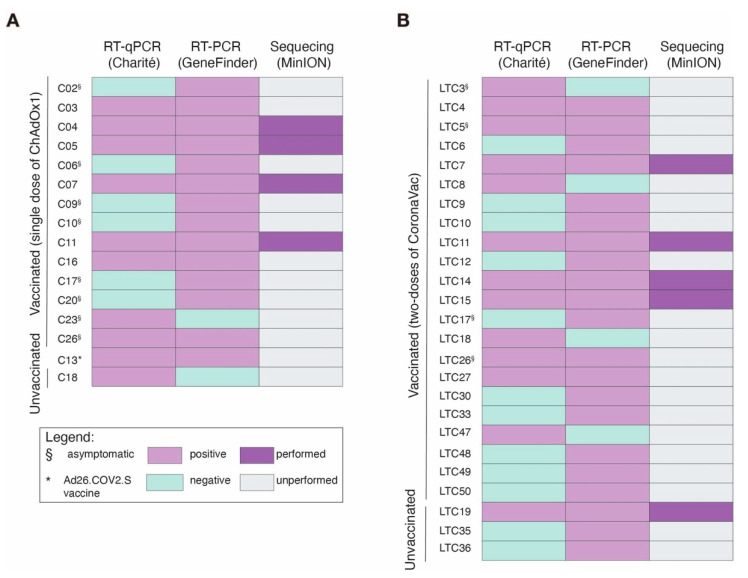
Diagnosis of SARS-CoV-2 cases from outbreaks described in this study. Outbreak A in convent (**A**), and outbreak B in long-term care facility (**B**). Abbreviations: RT-qPCR; real-time quantitative polymerase chain reaction; C, convent; LTC, long-term care facility.

Outbreak A occurred in a female convent, where 83.3% (15 of 18) of residents (i.e., nuns) and 87.5% (7 of 8) of employees were vaccinated with a single dose of ChAdOx1 vaccine on 5 February 2021. One resident was immunized with the Ad26.COV2.S (Janssen Pharmaceutical Companies, Beerse, Belgium) vaccine on 17 March 2021, during the phase 3 trial in Brazil [36] (Figure 1A). Of these, 25 (96.2%) were women, and the median age was 73 years (interquartile range: 50–83). Between 1 and 22 March 2021, 10 nuns reported respiratory signs or symptoms (i.e., cough, dyspnea, and respiratory distress). On March 24, serum and nasopharyngeal swab samples were collected from 26 individuals in the convent (Figure 1A). We performed RT-qPCR testing of all residents and employees, and SARS-CoV-2 RNA was detected in nasopharyngeal samples of 63.6% (14 of 22) of individuals who had received a single dose of ChAdOx1, including nine nuns and one employee. Moreover, one unvaccinated staff member and a resident who was vaccinated with Ad26.COV2.S were PCR-positive for SARS-CoV-2 RNA (Figure 2A). The interval between the vaccination and the onset of symptoms was at least 23 days. The median viral load was 3.4 × 10^1^ RNA copies per mL (interquartile range: 8.8 × 10^1^ to 4.3 × 10^3^) (Figure S1A). Overall, 50% (eight of 16) of cases were classified as mild COVID-19, and the other half were asymptomatic. None of the cases had moderate or severe COVID-19, and hospitalization was not required. According to the institution’s record, eight nuns had been outside the convent in the past 30 days, and all employees were living outside the convent.

Outbreak B occurred in an LTC facility, where 88.9% (32 of 36) of the residents and 62.5% (10 of 16) of the employees were immunized with two doses of CoronaVac vaccine between the 7 February and 4 March 2021 (Figure 1B). In addition, one employee was vaccinated with ChAdOx1 on 21 January 2021. Of these, 78.8% (41 of 52) were women, and the median age was 77 years (interquartile range: 51–87). Between 5 and 27 March, 2021, seven residents and one employee reported respiratory symptoms. On 29 March, serum and nasopharyngeal swab samples were collected from 50 of the 52 individuals in the LTC facility (Figure 1B). The interval between the second dose and onset of symptoms varied between 5 and 27 days. Here, we also performed serial RT-qPCR testing samples of residents and employees of the LTC facility, and we detected SARS-CoV-2 RNA in nasopharyngeal samples of 52.4% (22 of 42) of individuals, including 18 residents and four employees, who received two doses of CoronaVac vaccine at least 5 days before the first case of outbreak B (Figure 2B). We also estimated the median viral load as 2.3 × 10^3^ RNA copies per mL (interquartile range: 8.8 × 10^2^ to 1.4 × 10^5^) (Figure S1A). Interestingly, most cases (75%, 18 of 22) were asymptomatic, and four were classified as mild COVID-19. Furthermore, we detected SARS-CoV-2 RNA in nasopharyngeal samples from three unvaccinated individuals who were asymptomatic (ID: LTC 19, LTC35, LTC36). In addition, one resident (ID: LTC20) had an onset of symptoms on 25 March 2021, 21 days after the second dose of CoronaVac. This patient, who was 84 years old with Alzheimer’s disease, was SARS-CoV-2-positive by RT-PCR, was hospitalized on March 28, and died of COVID-19 on 19 April 2021. Lastly, we compared the viral loads between the patients infected with SARS-CoV-2 who were vaccinated with ChAdOx1 (*n* = 10) versus CoronaVac (*n* = 11), but no significant differences were observed (Figure S1A).

**Figure 3:**
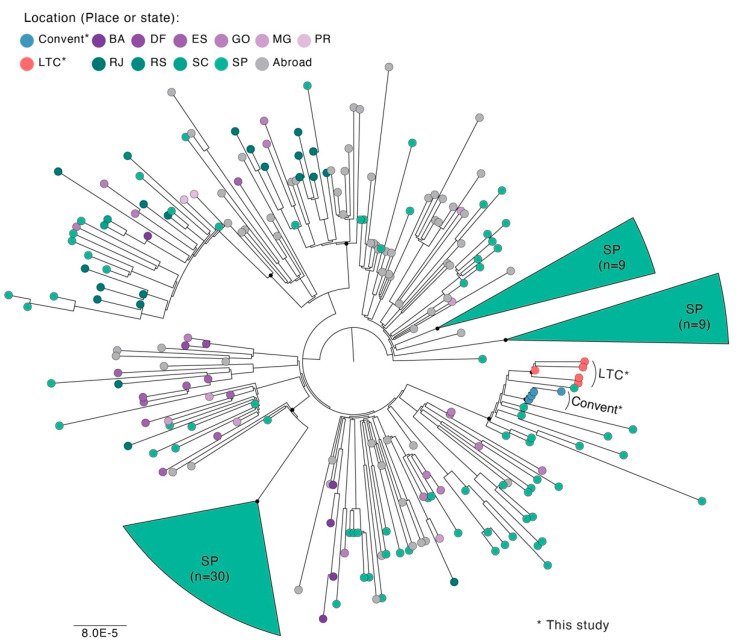
Maximum-likelihood tree (*n* = 264) was estimated using IQ-tree version 1.6.12 [27] and constructed using an alignment of the near-complete genome (untranslated regions were removed) and using a GTR + F + I substitution model [28] with 1000 ultrafast bootstrap replicates. The scale bar indicates the evolutionary distance in numbers of substitutions per nucleotide site. The color on terminal branches indicates the place (LTC facility, *n* = 5 and convent, *n* = 4) or Brazilian state reported, as indicated in the legend. The sequences are available on GISAID, accession numbers: EPI_ISL_2497923, EPI_ISL_2497906, EPI_ISL_2497898, EPI_ISL_2497896, EPI_ISL_2497882, EPI_ISL_2497881, EPI_ISL_2497867, EPI_ISL_2497849, and EPI_ISL_2497847; https://www.gisaid.org, accessed on 26 June 2021). Abbreviations: LTC, long-term care facility; BA, Bahia state; DF, Distrito Federal; ES, Espírito Santo state, GO, Goiás state; MG, Minas Gerais state; PR, Paraná state; RJ, Rio de Janeiro state; RS, Rio Grande do Sul; SC, Santa Catarina state; SE, Sergipe state; SP, São Paulo state; abroad, countries other than Brazil. Black dots indicate the main nodes with ML bootstrap support levels > 75% based on 1000 bootstrap replicates.

To investigate the SARS-CoV-2 lineage associated with these infections, we sequenced SARS-CoV-2 genomes from a subset of nasopharyngeal samples with RT-qPCR Ct-values ≤ 30 (outbreak A = 4 and outbreak B = 5). We obtained sequences with 78.6% to 97% genome coverage (minimum depth ≥ 20-fold) from these nine individuals. All SARS-CoV-2 genomes obtained from both outbreaks were classified into the B.1.1.7 (recently designated the alpha lineage according to a World Health Organization recommendation), and we confirmed all signature mutations from this lineage (Table S3). The ML tree revealed that the genome sequences obtained from outbreaks A and B clustered in independent clades (bootstrap values ≥ 99), suggesting two distinct transmission clusters (Figure 3). Furthermore, we found only one nucleotide difference among the SARS-CoV-2 genomes from outbreak A (convent), ≤2 nucleotide differences among the genomes from outbreak B (LCT facility), and six difference among the genome sequences from outbreaks A and B (Table S3). All sequences are available on GISAID (accession numbers: EPI_ISL_2497923, EPI_ISL_2497906, EPI_ISL_2497898, EPI_ISL_2497896, EPI_ISL_2497882, EPI_ISL_2497881, EPI_ISL_2497867, EPI_ISL_2497849, and EPI_ISL_2497847; https://www.gisaid.org, accessed on 26 June 2021).

We found that 95.5% (21 of 22) and 83.3% (35 of 42) of the individuals who received a single dose of ChAdOx1 or two doses of CoronaVac, respectively, were positive for IgG (S-RBD) antibody (cutoff ≥ 50.0 AU/mL). In addition, 25% (three of 12) of nonvaccinated patients were IgG (S-RBD)-positive, one was PCR-positive, and two were PCR-negative (Figure S1B). Patients who were SARS-CoV-2-infected and immunized with ChAdOx1 presented the higher median IgG antibody titer against spike protein (6687 AU/mL), which was statistically different compared to IgG (S-RBD) levels of unvaccinated individuals (*p*-value = 0.0165 and 0.00237 for PCR-positive and -negative, respectively). On the other hand, the detected immunoglobulin antibody levels elicited against the SARS-CoV-2 nucleocapsid protein in 18.2% (four of 22) individuals vaccinated with ChAdOx1 vaccine indicated a recent SARS-CoV-2 infection because this protein is not expressed from this vaccine (Table S1).

**Figure 4:**
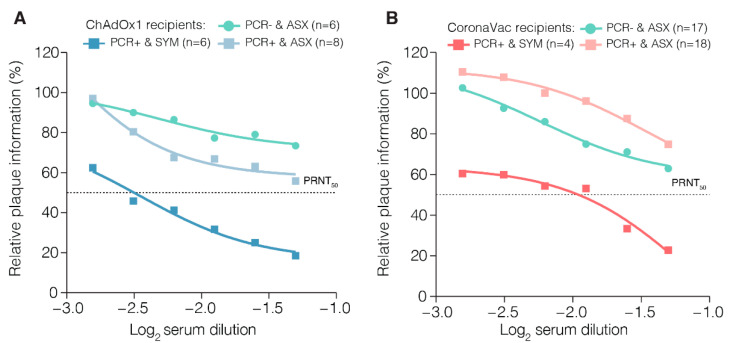
Neutralizing antibodies of sera from individuals vaccinated with ChAdOx1 and CoronaVac against B.1.1.7 isolate. (**A**) Sera from individuals from outbreak A in the convent (*n* = 20). (**B**) Sera from individuals from outbreak B in the long-term care facility (*n* = 39). The average value in log_2_ dilutions of the tested sera are shown. PRNT_50_ represents the dilution that showed a 50% reduction in plaque formation in comparison with a control without serum after three-parameter nonlinear regression analysis. The symbols represent the average of sera samples. Abbreviations: SYM, symptomatic. ASX, asymptomatic. PCR+, PCR-positive. PCR−, PCR-negative.

To measure the neutralizing antibody capacity in individuals infected with B.1.1.7 and previously vaccinated with a single dose of ChAdOx1 or two doses of CoronaVac, we isolated the B.1.1.7 lineage virus in Vero cells from a nasopharyngeal sample (ID: LTC7), and the isolate was designated B.1.1.7/LTC7. Cytopathic effects (CPE) in the Vero cell monolayer were observed 5 days post inoculation. Then, the cell culture supernatant was harvested, and the presence of SARS-CoV-2 RNA was confirmed by RT-qPCR targeting the envelope gene. The RT-qPCR protocol was used to confirm virus isolation through Ct values dropping from 22.5 (original sample) to 12.3 (first passage) and 12.5 (second passage). The titer of the B.1.1.7/LTC7 was 1.7 × 10^4^ PFU/mL (first passage) and 1.5 × 10^6^ PFU/mL (second passage). In addition, we confirmed the expected mutations by genome sequencing with coverage of 95.1% (first passage) and 92.6% (second passage) (Table S3). First and second passage sequences are available on GISAID with accession IDs EPI_ISL_2498612 and EPI_ISL_2498894. Subsequently, we tested the sera of individuals from the convent and the LCT facility by PRNT_50_ assays against the B.1.1.7 isolate. Sera from symptomatic individuals and qPCR-positives who received a single dose of ChAdOx1 (*n* = 6) had ~3.4-fold higher neutralizing titers against the B.1.1.7 lineage isolate compared to sera of asymptomatic ChAdOx1-vaccinated individuals (*n* = 6) (Figure 4A). The PRNT_50_ value for the B.1.1.7 isolate was ~1:80 in the sera of qPCR-positive individuals with mild COVID-19 who received two doses of CoronaVac (*n* = 17), whereas asymptomatic persons vaccinated with CoronaVac (*n* = 4) had PRNT_50_ values >1:20 (*n* = 4) (Figure 4B).

## 4. Discussion

We report two independent outbreaks involving local transmission of variant B.1.1.7 in individuals immunized with a single dose of an adenovirus-vectored COVID-19 vaccine or two doses of inactivated vaccine. Our data suggest that the B.1.1.7 lineage can infect individuals partially immunized with a single dose of ChAdOx1 or fully immunized with two doses of CoronaVac vaccine. Previous in vitro studies reported a reduction of 2.1–3.3-fold in the neutralizing titers of serum or plasma of immunized individuals (BNT162b2 or ChAdOx1 vaccines) against a B.1.1.7 isolate, compared to other SARS-CoV-2 lineages [6]. Plasma of CoronaVac-vaccinated persons after a single or two doses presented a lower neutralization capacity against P.1 isolates, another VOC that emerged and become the dominant lineage in Brazil [33]. In contrast, another study shows that plasma from individuals immunized with two doses of CoronaVac presents an effective neutralizing capacity against the B.1.1.7 lineage [37]. Here, we observed higher neutralizing antibody levels in sera from vaccinated individuals infected with the B.1.1.7 lineage compared with vaccinated but uninfected persons, which might be a consequence of immune restimulation, promoting the expansion of memory B cells and enhancing IgG production, as well as a polyclonal stimulus for ChAdOx1 recipients [38]. In addition, we observed higher antibody levels in vaccinated, PCR-positive, symptomatic individuals than in vaccinated, asymptomatic, PCR-positive individuals, indicating that the neutralizing antibody levels may not reliably predict protection against mild COVID-19. In fact, other components of the immunological response are probably essential for SARS-CoV-2 restriction, such as T-cell-mediated immunity, which plays a crucial role in controlling SARS-CoV-2 infection [39].

For most individuals involved in the two outbreaks we studied, the transmission of infection was accounted for by close contact during prolonged periods, providing an opportunity for viral transmission. These social interactions are similar to large family gatherings, schools, workplaces, and churches [40,41,42]. To mitigate the impact in both outbreaks, several clinical and epidemiological measures were adopted, such as the protocol of use of personal protection equipment by care workers and resident people was reinforced; the number of people was limited by physical distancing in specific rooms and places of common use; daily monitoring of eventual symptomatic among care workers and/or resident people was performed. In addition, an active investigation was conducted to identify potential additional suspected cases and contact tracing. There were established different cohorts of confirmed cases, suspected cases, asymptomatic contacts, and the noncontact asymptomatic population; each group was isolated under specific conditions, such as isolation during the infective period or isolation while waiting for laboratorial results or quarantine during incubation period, always in different areas and sectors of their respective institutions. Respiratory samples for testing by RT-PCR were collected from every symptomatic respiratory case, including care workers, resident people, and their respective contacts during the outbreak’s follow-up period. Visits from external people did not become authorized until the outbreaks were considered controlled. Based on phylogenetic analysis, our findings support that the outbreaks reported here were caused by two independent B.1.1.7 lineage transmission clusters. Alternatively, we cannot exclude the possibility that other lineages infected the other cases that were not sequenced, owing to the higher prevalence of the gamma variant at the time [43]. However, during the clusters of SARS-CoV-2 caused by lineage B.1.1.7 in this study, the variants of interest such as lambda and mu, as well as the delta VOC, were not described in Brazil.

The elderly have a higher risk of hospitalization and fatal COVID-19 cases [44,45]. Furthermore, infection with the B.1.1.7 lineage has been associated in some studies with increased risk of COVID-19 hospitalization and death [3,4]. Although the median age of vaccinated individuals in both clusters was between 73 and 77, 23 individuals were asymptomatic and 10 experienced mild COVID-19. Similarly, a previous study in the UK with participants aged ≥70 years and vaccinated with a single dose of BNT162b2 or ChAdOx1 showed the association of vaccination with a significant reduction in symptomatic disease and severity COVID-19 [46]. Another study in an LTC facility reported that a single dose of BNT162b2 did not prevent severe or fatal COVID-19 in the elderly (median age = 88 years old) 3 weeks after the initial vaccination [47]. We report here the first fatal COVID-19 case in an 84 year old individual who received the complete series of CoronaVac vaccination. Overall, our data suggest protective effects, against severe COVID-19 and death caused by the B.1.1.7 lineage, of a single dose of ChAdOx1 or two doses of CoronaVac.

A previous study in the UK reported that 90.15% of people who received the ChAdOx1 vaccine developed IgG antibodies within 3 weeks of the first dose [48]. In addition, the IgG seroconversion in the CoronaVac phase 1/2 clinical trial was between 83% and 100% depending on immunization dose [12]. Here, we reported similar findings with IgG (S-RDB) seroconversion of 95.5% and 83.3% of individuals immunized with the ChAdOx1 and CoronaVac vaccines, respectively. Since ChAdOx1 recipients develop humoral responses against the spike protein, the detection of total immunoglobulins specific against the SARS-CoV-2 nucleocapsid in ChAdOx1 recipients indicates SARS-CoV-2 infection. On the other hand, we cannot serologically access the SARS-CoV-2 infection in CoronaVac recipients because this inactivated vaccine promotes a polyclonal response to both the spike and the nucleocapsid proteins, which impairs our ability to differentiate vaccinated from infected patients using serological assays [12]. In addition, the primary efficacy against symptomatic COVID-19 was 66.7% for ChAdOx1 and 50.7% for CoronaVac, both involving a two-dose regimen [49,50]. In the two clusters presented here, we observed symptomatic SARS-CoV-2 cases in 42.9% (six of 14) and 22.7% (five of 22) of individuals vaccinated with a single dose of ChAdOx1 and two doses of CoronaVac, respectively. Despite the important limitations, such as small size, limited sites, and individuals who belong to institutional settings and who are not community dwellers, these data suggest that the efficacy of these vaccines against severity and mortality is maintained outside the clinical trial setting.

## 5. Conclusions

In conclusion, our observational data based on two outbreaks with small sample size suggest that the SARS-CoV-2 B.1.1.7 lineage can infect individuals partially immune with a single dose of adenovirus-vectored or fully immunized with two doses of inactivated vaccines. Furthermore, many people remain susceptible to infection and probably contribute to chains of transmission through viral shedding. However, partial immunization with ChAdOx1 or the complete series of CoronaVac has some protective effect against severity and death caused by COVID-19. Therefore, these data underscore the critical importance of continued and rapid immunization to reduce the burden of COVID-19 in Brazil. Lastly, nonpharmaceutical interventions (e.g., masking and physical distancing), even in vaccinated persons, are needed to interrupt the SARS-CoV-2 transmission cycle until collective immunity is improved through vaccination.

## Data Availability

Standard R-packages or Prism software were used to produce results for this analysis. No custom code was developed.

## Supplementary Materials

The following are available online at https://www.mdpi.com/article/10.3390/v13112127/s1: Figure S1. SARS-CoV-2 viral load and IgG (S-RDB) antibody levels from outbreaks reported in this study; Table S1. Information of individuals from SARS-CoV-2 outbreaks in the convent and the long-term care facility; Table S2. GISAID ID, authors, and submitting laboratory; Table S3. Information of genome sequencing of SARS-CoV-2 lineage B.1.1.7 from SARS-CoV-2 outbreaks in convent and a long-term care facility, and B.1.1.7 isolate used in neutralizing antibody assays; Table S4. Single-nucleotide polymorphism for B.1.1.7 from SARS-CoV-2 outbreaks in convent and a long-term care facility.
